# Mapping bundles of ecosystem services reveals distinct types of multifunctionality within a Swedish landscape

**DOI:** 10.1007/s13280-014-0601-0

**Published:** 2015-01-09

**Authors:** Cibele Queiroz, Megan Meacham, Kristina Richter, Albert V. Norström, Erik Andersson, Jon Norberg, Garry Peterson

**Affiliations:** Stockholm Resilience Centre, Stockholm University, Kräftriket 2B, 106-91 Stockholm, Sweden

**Keywords:** Ecosystem services, ES bundles, Trade-offs, Synergies, Regional scale, Landscape management

## Abstract

Ecosystem services (ES) is a valuable concept to be used in the planning and management of social–ecological landscapes. However, the understanding of the determinant factors affecting the interaction between services in the form of synergies or trade-offs is still limited. We assessed the production of 16 ES across 62 municipalities in the Norrström drainage basin in Sweden. We combined GIS data with publically available information for quantifying and mapping the distribution of services. Additionally, we calculated the diversity of ES for each municipality and used correlations and k-means clustering analyses to assess the existence of ES bundles. We found five distinct types of bundles of ES spatially agglomerated in the landscape that could be explained by regional social and ecological gradients. Human-dominated landscapes were highly multifunctional in our study area and urban densely populated areas were hotspots of cultural services.

**Electronic supplementary material:**

The online version of this article (doi:10.1007/s13280-014-0601-0) contains supplementary material, which is available to authorized users.

## Introduction

Ecosystem services (ES) are the benefits provided by ecosystems to humans and are often distinguished as provisioning, regulating, cultural, and supporting services (MA 2005). These services are often co-produced by humans and nature having therefore an intrinsically social–ecological character (Andersson et al. 2007; Reyers et al. 2013). As ES have direct and indirect impacts on human well-being, they are a valuable tool to be used in landscape planning and management, and when communicating the value of nature to different stakeholder groups and decision makers (Kareiva et al. 2011).

While ES research has increased exponentially over the past decade, the assessment and measurement of these services are still challenging (Tallis and Polasky 2011). So far, most research in this area has been developed for data-rich situations or required investment in substantial data collection. Furthermore, with the exception of provisioning services, most ES are difficult to quantify (Turner and Daily 2008; Norris 2012). The flow and delivery of individual services depend on both social factors, such as policies, management practices, or human demand, and biophysical processes (de Groot et al. 2002). This complexity confounds ES assessments and has led to calls for new empirically based research in this field (e.g., Carpenter et al. 2006; Daily and Matson 2008; Johnson et al. 2012). Specifically, there is a need for social–ecological integration and lower cost assessments of ES.

In addition to the social–ecological complexity inherent to the generation of individual ES, these also interact with each other in often unpredictable ways (Rodríguez et al. 2006). Some services “come together” in interdependent bundles, while others occur as trade-offs (Bennett et al. 2009; Raudsepp-Hearne et al. 2010). Trade-offs can also occur across space and time, meaning that the increase in the provision of a particular service may negatively affect ES elsewhere or in the future (Rodríguez et al. 2006; Wang and Fu 2013). Additionally, observed trade-offs between two services can be due to the effect of a common driver or a real interaction between those services. For example, the use of fertilizers and pesticides for improving crop yields is a driver that leads to trade-offs between crop production and water quality. In contrast, the trade-off between carbon sequestration and water provisioning is the result of a real interaction between services, as the increase in evapotranspiration caused by tree growth decreases water availability (Bennett et al. 2009). Thus, depending on the type of trade-offs, different measures will be required for mitigating negative outcomes.

A growing number of studies have conducted empirical assessments of multiple ES and their interactions (e.g., Lavorel et al. 2010; Raudsepp-Hearne et al. 2010; Andersson et al. 2012; Maes et al. 2012; Martín-López et al. 2012; Plieninger et al. 2013; Qiu and Turner 2013; Turner et al. 2014). However, not surprisingly, most of this research varies in scale and methodology, which makes cross-study comparisons difficult. Comparisons across studies are important in ES research, because they allow for the generalization of local or regional findings and help to disentangle the effect of context-dependent drivers from real interactions between services.

This paper aims to contribute with a case-based empirical approach that can easily be comparable to other case studies. We follow the approach developed by Raudsepp-Hearne et al. (2010) and replicated by Turner et al. (2014) to examine the existence of bundles of ES across the Norrström drainage basin in the Stockholm region. Numerous studies have focused on the social processes and practices behind the generation of ES in this region (e.g., Andersson et al. 2007; Ernstson et al. 2008; Barthel et al. 2010; Ernstson et al. 2010; Andersson et al. 2015), and have highlighted how urban spaces can provide multiple services. However, most of these studies have focused explicitly on the city of Stockholm and its neighboring municipalities. Additionally, with one exception (Andersson et al. 2015), none of them specifically aimed to assess and quantify bundles of multiple ES, as we propose to do in this paper. The article by Andersson et al. (2015) differs from our study as it explores the farm to landscape scale, meaning also that the set of services and their indicators were different from the ones in this paper.

Our paper has four main goals as follows: (a) to identify patterns of multiple ES across a diverse landscape, (b) to characterize the type and strength of interactions among both individual services and bundles of ES, (c) to investigate how the bundling of these services compares to that found in other regions, and (d) to discuss the implications of the different bundles of ES for landscape management in the Norrström region, and other regions alike.

We consider that the results originated by our study can provide valuable comparative insights that can contribute to the better understanding of local and global patterns of interactions between services and be useful for local and regional stakeholders, managers, and decision makers in the Norrström region.

## Materials and methods

### Study area

**Fig. 1:**
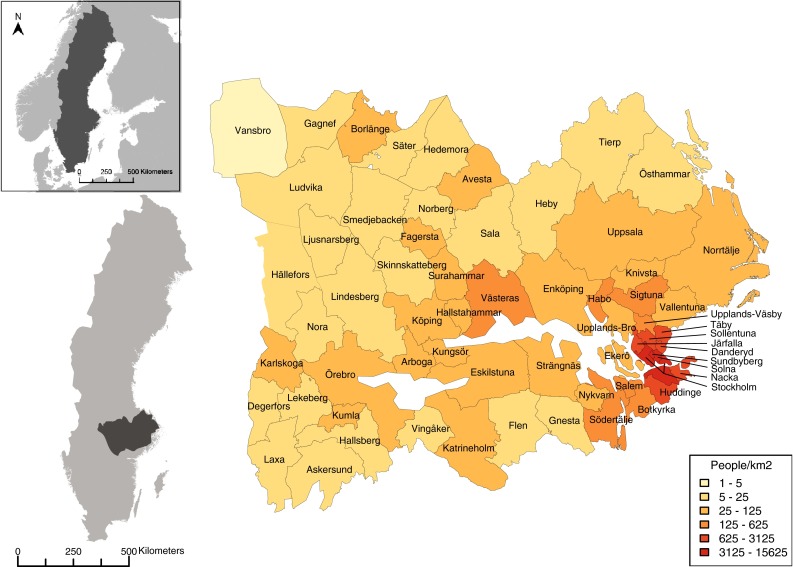
Location of the study area in the Norrström drainage basin, Sweden, and the 62 municipalities across the basin. The *colors* in the Norrström basin map indicate the population density in each municipality

The Norrström drainage basin is situated in south-central Sweden and covers 22 650 km^2^ (Fig. 1). The area has an east–west climate gradient from maritime in the south-east to continental in the north-west. Rainfall is higher during summer months (up to 60 mm day^−1^), than in winter (up to 25 mm day^−1^), summing up a total of 500–600 mm per year. The basin is heterogeneous in terms of land cover and land uses, usually with a mix of land uses at a fine scale, and includes two of Sweden’s largest lakes, Lake Mälaren and Lake Hjälmaren. Agricultural land is dominant (primarily pasturage, cereal production, and rapeseed) on the near surroundings of lake Mälaren, while the north-west is, to a large extent, dominated by production forest (primarily Norway Spruce *Picea abies*). The eastern part of the region is heavily influenced by the city of Stockholm and its extended metropolitan area, with high population density (Sporrong 2008). Food production landscapes have undergone major changes since the 1950s, either through reforestation and a shift away from full-time family farming or development toward larger farm units and intensified use. The water areas in the Norrström basin provide several important ES. Lake Mälaren, for example, is the main regional water supply for more than 1.5 million people in the Stockholm region. The Norrström basin is also famous for salmon fishing, and the surroundings of Lake Mälaren are known for their high natural and cultural values (including outdoor recreation).

### Data collection

We assessed 16 ES (six provisioning services, five regulating services, and five cultural services) in 62 municipalities within the Norrström basin (Table 1; Fig. 1). We focused mainly on services provided by terrestrial ecosystems and inland waters, despite some of these were also significantly affected by the proximity to marine and coastal areas. We did not include supporting services in our analysis as these provide some fundamental functions to other types of services and therefore, we assumed their expression would be captured by other services assessed. Also, the classification of supporting services is controversial and it has been argued that these should rather be classified as “ecosystem functions” underlying the production of provisioning, regulating, and cultural services (e.g., TEEB 2010).

**Table 1:** List of ES assessed in this study, indicators and respective units for individual services, and data sources

Ecosystem services	Indicator	Units	Source
Provisioning			
Wheat production	Wheat cultivated area	km^2^	Swedish Board of Agriculture National Statistical Database
Cattle	Number of animals by agricultural holding	Cattle/km^2^	Swedish Board of Agriculture National Statistical Database
Pig production	Number of animals by agricultural holding	Pigs/km^2^	Swedish Board of Agriculture National Statistical Database
Sheep	Number of animals by agricultural holding	Sheep/km^2^	Swedish Board of Agriculture National Statistical Database
Forest products	Commercial forest area	km^2^	National Land Survey and National Board of Forest
Regulating			
Crop pollination	Amount of pollinator habitat (specified on supplementary material) within a buffer of 200 m from crop production areas	km^2^	Swedish Land-Cover Data (*Marktäckedata*), based on Corine land cover 2006
N retention	Amount of nitrogen retained by the soil from the total load	Average fraction of 1-(Net nutrient load/Gross nutrient load)	Swedish Environmental Emissions Data (SMED)
P retention	Amount of phosphorus retained by the soil from the total load	Average fraction of 1-(Net nutrient load/Gross nutrient load)	Swedish Environmental Emissions Data (SMED)
Standing water quality	Ecological status of the water (classes 1–5, from low to high quality)	Average water quality/km^2^	Water information system Sweden (VISS) database
Running water quality	Ecological status of the water (classes 1–5, from low to high quality)	Average water quality/km^2^	Water information system Sweden (VISS) database
Cultural			
Moose hunting^a^	Number of moose shootings reported in hunting areas	Number of animals shot/km^2^	Swedish County Administrative Board
Outdoor recreation	Total amount of area defined as high interest for outdoor recreation by the Swedish Nature Protection Agency according with the Swedish Environmental Law	km^2^	Swedish County Administrative Board
Summer cottages	Total area of second habitation recreational areas	km^2^	Swedish National Statistics database (SCB)
Horseback riding	Number of horses by agricultural holding	Horses/km^2^	Swedish Board of Agriculture National Statistical Database
Cross-country skiing	Number of ski stations with prepared tracks	Stations/km^2^	Swedish ski tracks online database
Biodiversity appreciation	Number of species reported in each municipality	Species/km^2^	Species observations report database *Artportalen*. Report system for plants, animals and fungi

^a^Moose hunting is also a provisioning service, as it provides wild meat. However, the recreational economical value of hunting is in Sweden almost two times higher than the meat value (64 against 36 % of the total hunting value, respectively) (Mattsson et al. 2009). Therefore in this study, we considered moose hunting as a cultural service

Municipalities ranged in area from 8.77 to 2234.47 km^2^, and averaged 574.19 km^2^. We chose the municipality scale, as this is the smallest scale of governance in Sweden, at which many decisions regarding planning and landscape management are taken. Additionally, this is an intermediate landscape scale that captures both the variation of services with a regional or global expression, such as carbon sequestration or water quality regulation, and others locally produced, such as crop and livestock production and some cultural services.

We used three main criteria for ES selection: (1) the service had to be identified in previous studies and/or public data sources as important to the users in the Norrström region, (2) data on potential indicators for the service had to be available at the municipality level, (3) as we wanted to assess interactions between services, the total range of services should include a balanced share of each ES category (similar number of provisioning, regulating, and cultural services).

Based on these criteria, we identified a broad range of indicators. Most of these were indicators of the potential for the production of a particular service. Still, for cultural services and some provisioning services, we used indicators related to the use of each service (Table 1). Indicator data were retrieved from public databases from the national governmental authorities or other national and regional institutions (Table 1). In situations where multiple indicators for the same ES were found, we favored those that were publically available, exhibited a greater amount of variation among municipalities and were independent of other indicators (see 10.1007/s13280-014-0601-y for details).

### Data analysis

To enable comparisons across municipalities, indicators for each service were divided by municipality area (land area for all services except outdoor recreation, which included both land and water areas; and water quality data which were divided by total water area assessed in each municipality). Measurements obtained for each service were further normalized by the maximum value for that particular service, to facilitate comparisons of interactions among services.

We used the QGIS 2.0.1-Dufour Geographical Information Systems to produce maps of the distribution of individual services across the basin, and all data analyses were conducted in R (R Core Team 2013). As most relationships between pairs of services in the study area, and at this scale of analysis, could be approximated to a linear function (Fig. 10.1007/s13280-014-0601-y, Electronic Supplementary Material), we used correlation analysis to identify the existence of synergies and trade-offs. We did not aim to quantify every specific trade-off or synergy function for each pair of services. Rather, this was a first step in identifying weak and strong relationships among multiple services that could indicate the existence of a synergy or trade-off between two services. Therefore, in the context of this paper, we will refer to trade-offs or synergies, whenever negative or positive significant relationships between two services have been identified by the correlation analysis.

We categorized ES in provisioning, regulating, and cultural (Table 1) and mapped the average values for each category across the municipalities. Additionally, we produced maps of the residuals from the regional average, to identify “hot” (higher production) and “cold” (lower production) municipalities for each category of services. We mapped the multifunctionality of each municipality by calculating a derivation of the original Simpson’s index of the level of ES in each municipality, where higher values corresponded to higher diversity (see 10.1007/s13280-014-0601-y for details). While this index is traditionally used to estimate biodiversity, it has been applied to estimate the diversity of ES in previous studies (Raudsepp-Hearne et al. 2010). As all services were present in the 62 municipalities, we considered that municipalities with higher evenness across services were more diverse than those with an uneven distribution of services. Furthermore, we tested whether the supply of regulating services was related to the diversity of non-regulating ES in each municipality, as it has been found elsewhere.

We assessed the interactions among ES using Principal Component Analysis (PCA) and cluster analysis. We used a PCA to identify the main explanatory factors of the variability and distribution of the 16 ES across the municipalities. *K*-means clustering was used to identify distinct types of bundles of ecosystems. We compared these clusters to those produced using model based clustering and selected the number of clusters based on cluster robustness and explained variance. We used rose-wind diagrams to visualize ES bundles. These diagrams were dimensionless, as all bundles were calculated from the normalized values obtained for each service. Figures were created using the R packages maptools (Bivand 2013) and RColorBrewer (Neuwirth 2011). Cluster analysis was done using the R packages vegan (Oksanen et al. 2013), mclust (Fraley et al. 2012), and fpc (Hennig 2013).

## Results

### General patterns for ES distribution across the basin

**Fig. 2:**
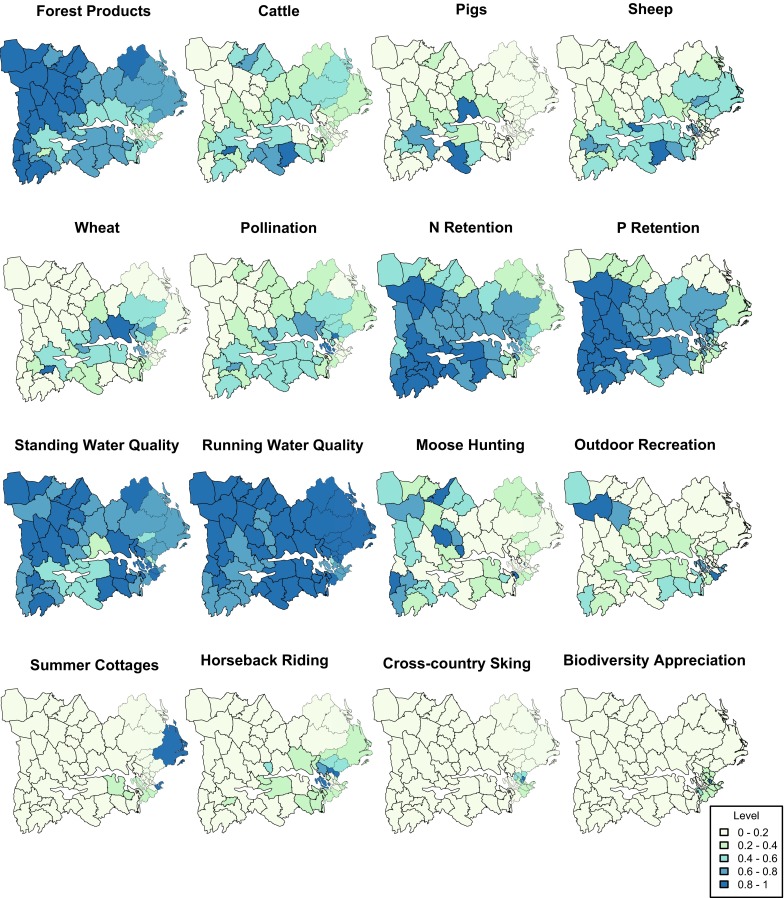
Distribution of the values obtained for individual ES across the 62 municipalities in the Norrström drainage basin. *Darker shadows of blue* represent a higher production of the service

The distribution of provisioning, regulating, and cultural services varied substantially across the basin (Fig. 2). Regulating services were the most evenly distributed, while cultural services were highly clustered around Stockholm. With the exception of forest products, provisioning services were mainly concentrated around lakes Mälaren and Hjälmaren. Due to its connection to agriculture, crop pollination was the one regulating service also concentrated around these regions (Fig. 2). Moose hunting and outdoor recreations were the only cultural services not centered on Stockholm.

### Interactions between services

We found strong negative and positive interactions among many of the ES assessed (Fig. 10.1007/s13280-014-0601-y, Electronic Supplementary Material). Of 120 possible pairs of services, 23 were significantly negatively correlated and 25 were significantly positively correlated. Cross-country ski, biodiversity appreciation, moose hunting, and forest products were significantly negatively correlated with a high number of services (seven, six, five and five, respectively). Biodiversity appreciation and cross-country ski were concentrated in urban areas, while forest products and moose hunting were associated with forested areas. High number of positive interactions was found for crop pollination, wheat and livestock production (significantly positively correlated with five, five and four services, respectively) and these services were typically associated with agricultural landscapes. Thus, we found more trade-offs between services in both urban and relatively dense forested areas, than in agricultural areas.

**Fig. 3:**
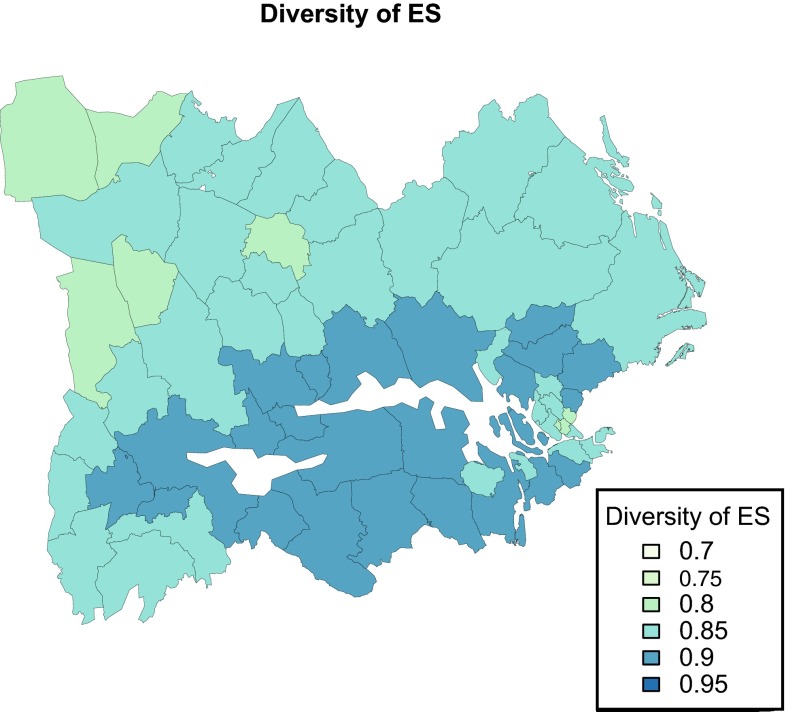
Diversity of ES across the 62 municipalities. Diversity was calculated by a derivation of the original *Simpson’s diversity index* and was higher on municipalities with an even distribution of services

Most municipalities produced a varied set of ES, and diversity was high across the entire region (Fig. 3). Lowest diversity values were presented in some urban municipalities of the Stockholm region (Sundbyberg, Solna, and Danderyd) and some rural municipalities mainly dominated by production forest (Gagnef, Hällefors, Ljusnarberg, Norberg, and Vansbro). This is in agreement with the trade-offs described above between (i) cultural services in the Stockholm area and other regulating and provisioning services and (ii) forest-related services (moose hunting and forest products) and other types of services. We found a weak relationship (*R* = 0.23, *p* = 0.07) between the supply of regulating services and the diversity of provisioning and cultural services.

**Fig. 4:**
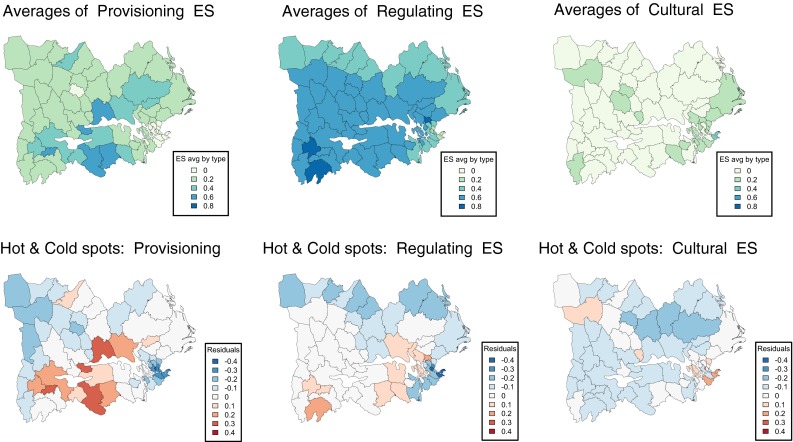
Hot and cold spots for types of ES (provisioning, regulating, and cultural) across the study area (lower three figures). Hot spots (represented by an increasing gradient of *red*) are municipalities with particularly high production of a given type of service, while cold spots (represented by a decreasing gradient of *blue*) are municipalities where the production of a given type of service is particularly low. Average values of the three ES categories are shown in the upper three figures

With some exceptions, the municipalities around lakes Mälaren and Hjälmaren had the highest services’ diversity. Many of these municipalities were also identified as hot spots for the production of provisioning services (Fig. 4). This was corroborated by the synergies found between agricultural provisioning services and some regulating services (crop pollination, P retention), highlighted by the correlation analysis (Fig. 10.1007/s13280-014-0601-y, Electronic Supplementary Material). Despite the overall high diversity of services across the study area, hot and cold spots of provisioning, regulating, and cultural services were correlated with one another in ways that suggested the existence of trade-offs and synergies between these different categories. In particular, the hottest spots of one category were generally cold spots for the other categories. For example, the municipality of Västerås, identified as the hottest spot for provisioning services, was a cold spot for both regulating and cultural services.

### Bundles of ES

The PCA (Fig. 10.1007/s13280-014-0601-y, Electronic Supplementary Material) identified two gradients that explained most of the data variability. The first, explaining 26 % of the variance, was a gradient of population density from low to high, where densely populated urban municipalities had higher concentration of cultural services. The second (explaining 22 % of the variance) represented a land-cover gradient from forests to agricultural land, separating forest-related services (water quality, moose hunting, forest products) from those associated with agriculture (wheat, cattle, pig and sheep production, pollination, and horseback riding).

**Fig. 5:**
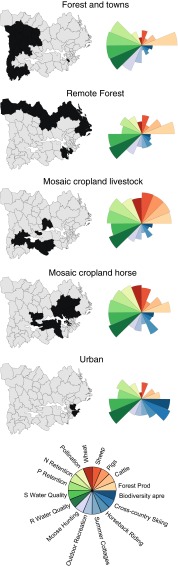
Bundles of ES identified by *k*-means clustering for the study area. The five groups of bundles (on the *right side of the figure*) are represented by rose-wind diagrams and named according to their characteristics. The diagrams are dimensionless, as they are based on normalized data for each service, and a higher surface area indicates the higher production of a particular service. The municipalities included in each group of bundles are highlighted in *dark gray* on the maps located at the *left side of the figure*

**Fig. 6:**
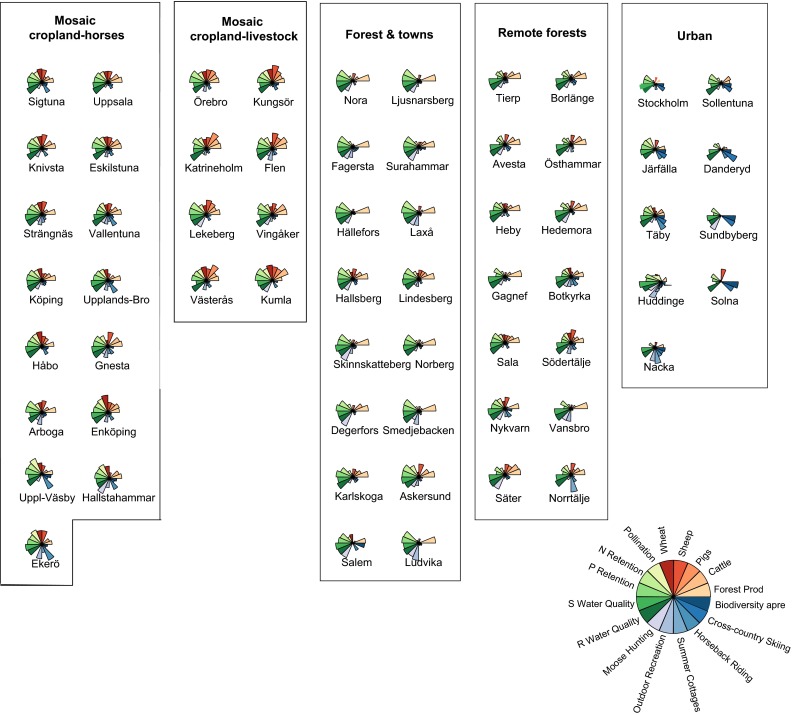
Bundles of ES found for each municipality. Municipalities are organised inside each bundles group (delimited by a rectangular area) from top to down according to their representativeness of that same group. Hence, municipalities at the top edge of the bundle group are more typical of that respective group than municipalities further down

We found five main groups of bundles of ES in the Norrström basin, and these were spatially clustered in the landscape (Fig. 5). The number of municipalities present in each group varied from 8 to 16 (Fig. 6). We named these bundle groups based upon the dominant ES and land cover, according to our knowledge of the municipalities within each cluster:

#### Mosaic cropland-horses

Regulating services, wheat and sheep production, provisioning of forest products and horseback riding were high in the *mosaic cropland*-*horses* group (*n* = 15). In contrast, pig production, summer cottages, cross-country ski, and biodiversity appreciation had low relative values in this group. The municipalities in this group were mainly located around lake Mälaren.

#### Mosaic cropland livestock

The *mosaic cropland*-*livestock* group (*n* = 8) comprised most municipalities around lake Hjälmaren and a couple around lake Mälaren. This group was quite heterogeneous, combining high pig and livestock production, provision of forest products and fairly high crop production, with high regulating services. Nevertheless, all cultural services had a relatively low performance.

#### Forest and towns

This group (*n* = 16) included municipalities located on the western area of the basin. Forests used for commercial purposes, scattered by small towns, were dominant in this area, which was reflected by the high provision of forest products and moose hunting. All regulating services, except crop pollination, were high in this group while most provisioning services presented relatively low values.

#### Remote forests

*Remote forests* (*n* = 14) contained municipalities mainly located in the northern part of the basin, an area characterized by planted coniferous forests and high clay-silica soils. This group had relatively low production of most services except provision of forest products and water quality.

#### Urban

Finally, the *urban* group (*n* = 9) corresponded, as the name indicates, to urban municipalities of the Stockholm region. Cultural services had a high expression in this group, particularly cross-country skiing and biodiversity appreciation but also a fair amount of horseback riding. Water quality was also relatively high in urban municipalities, being the only service that presented a consistently high performance across the five bundles groups.

The *mosaic cropland*-*horses* and *mosaic cropland*-*livestock* groups were the ones with higher production of multiple services. The municipalities in these groups were located in regions with a high diversity of land uses, from water bodies to mixed low-intensity agriculture and forested patches (Sporrong 2008). The groups *forest and towns*, *remote forests* and *urban* presented more trade-offs among services and these trade-offs were higher in *remote forests*.

Figure 6 represents the patterns of interaction among services obtained for each municipality, organized in their respective clusters or groups of bundles. Municipalities could be more or less typical of their bundle group and some less representative municipalities changed group whenever we did the cluster analysis for four or three clusters, instead of five.

## Discussion

We found significant differences on the patterns of ES distribution across the study area, with cultural services presenting the highest spatial aggregation.

The production of an ES is a combination of the social–ecological potential (ecological and biophysical conditions, management practices) of a landscape to produce it, and the human demand for that same service (Ernstson et al. 2010; Reyers et al. 2013). Cultural services were assessed through indicators that expressed the demand/use of a particular service. Therefore, the clustering of cultural services around Stockholm reflects a higher demand for this type of services in this region. Cultural services are often locally produced, as they result from the direct experience of nature by people. Thus, a greater expression of these services in higher population density areas, such as urban and peri-urban municipalities, is not surprising. Nevertheless, high population densities can also constrain ES, as the case of outdoor recreation and moose hunting, which were higher in lower populated forested areas elsewhere. This is due to both ecological factors, such as the lack of large connected semi-wild ecosystems in cities, as well as restrictions on hunting on urban areas.

This urban demand for cultural ES was found in previous work in Spain (Martín-López et al. 2012), and Danish peri-urban landscapes have been shown to be important for services such as recreation, sense of place, and nature appreciation (Turner et al. 2014). These findings are particularly interesting in the Swedish context, as the connection with nature and access to outdoor life is very imbedded in the Swedish culture and often expressed by people as essential to their well-being (Gidlöf-Gunnarsson and Öhrström 2007). For example, the possession of a summer cottage, is not only restricted to high-income people rather being spread at all society levels, and often meaning more than a holiday residence inserted in a resort area. Summer cottages give sense of place and open access to surrounding outdoor areas (often forest or water), where a wide range of popular activities can be experienced (forest walks and jogging, mushroom and berry picking, bird watching, swimming, boat riding, fishing, biodiversity appreciation, aesthetical beauty).

We found a variety of strong correlations among ES, and these reflected both similarities and dissimilarities with patterns found in other studies. The positive correlations found between forest products and regulating services (water quality and nutrient retention) reflect the synergies between forest areas and regulating services described in other work (MA 2005; Mäler et al. 2013). In contrast, the absence of strong negative trade-offs between provisioning agricultural services and other regulating and cultural services differed strikingly from studies using similar approaches in Montreal (Raudsepp-Hearne et al. 2010) and Denmark (Turner et al. 2014), as well as other studies (e.g., Maes et al. 2012). This might be explained by the relatively low intensity of agricultural practices in the Stockholm region, where agricultural areas hold a diverse number of habitats mixed in a mosaic-type landscape (Sporrong 2008). This differs from the Montreal region, dominated by high input intensive agriculture (Raudsepp-Hearne et al. 2010), and Denmark, which has the highest agricultural land use pressure in Europe (Turner et al. 2014).

Nevertheless, hotspots of provisioning services were cold spots of regulating services and vice versa, suggesting that trade-offs appear whenever a particular type of service is maximized. Still, the high ES diversity across the study area points for high multifunctionality at the municipal scale. Multifunctional landscapes have been correlated with higher levels of regulating services (Raudsepp-Hearne et al. 2010). However, we only found weak evidence for such a relationship. One explanation might be that most municipalities in our study had a high diversity of regulating services, which weakens the potential to detect such a relationship.

We found that bundles of services were associated to different types of social–ecological systems spatially aggregated across the basin. These patterns could be explained by social and ecological gradients in the landscape, and similar findings were found for other empirical analyses of multiple ES (Raudsepp-Hearne et al. 2010; Maes et al. 2012; Martín-López et al. 2012; Plieninger et al. 2013; Andersson et al. 2015). Distance from Stockholm strongly predicted the patterns found for most ES, while proximity to inland and coastal water was determinant for wheat production and summer cottages, respectively. This demonstrates how patterns of ES are shaped by the historical social–ecological interactions among people and nature.

Multifunctionality was the highest on the *mosaic cropland livestock* and the *mosaic cropland horse* bundle groups, but municipalities of the *urban* group could also provide multiple services. Swedish urban areas are relatively green, and there has been substantial historic public investment in urban green infrastructure. Therefore, we expect Stockholm to have relatively high levels of ES compared to other urban regions in other countries. Still, high multifunctionality and provision of cultural services in urban and peri-urban areas have also been found in Denmark (Turner et al. 2014). While both associated with forestry-dominated areas, and thus similar in land cover, the *remote forest* group and the *forest and towns* groups substantially differed on their capacity for nutrient retention. This might be explained by different factors in inland and coastal municipalities in *remote forests* that negatively affect nutrient retention. Due to their proximity to the Baltic, coastal municipalities have a short traveling distance of nutrients to the sea, which is the cause of low retention levels. Additionally, *remote forest’s* inland municipalities are crossed, from west to east, by the Dalälven River that quickly transports nutrients from inland to the Baltic Sea, not allowing for high nutrient retention (Fölster et al. 2012). In this case, biophysical and topographical characteristics of the landscape are key elements for understanding the interactions between services. This is a typical example where the influence of other factors can easily be interpreted as a real trade-off between services. Without knowing the geography of this region, the *remote forest* bundle group could easily be interpreted as a trade-off between water quality, production of forest goods, and nutrient retention when in reality the low nutrient retention level in these municipalities is not the result of a real negative interaction between these services.

As for individual services, the five bundles had both similarities and dissimilarities with the Montreal region (Raudsepp-Hearne et al. 2010) and Denmark (Turner et al. 2014). These contained municipalities dominated by intensive agriculture, destination tourism, and county homes, none of which were present in the in the Stockholm region. Nevertheless, similar bundle groups were identified in Montreal, despite the Swedish bundle group *urban* had higher levels of cultural services. Overall, our results suggest that the Montreal and Danish regions are more intensively used and less diverse within municipalities than the Stockholm region. Still, there are enough similarities among bundle types to suggest that the extension of the approach used in this paper to other places of the world could be useful to identify general patterns in which ES are co-produced.

We aimed to identify patterns of distribution of multiple ES and characterize the strength and type of interactions among services by using publicly available data at a scale that was relevant for governance. Therefore, we did not conduct our own field assessment and rather focused on already existing data, relevant for our study. While this methodology has clear advantages, allowing a cheap and rapid ES assessment in a given area, it also presents limitations. Firstly, the use of single indicators for individual services does not capture the full complexity of the interaction between the ecological potential, management practices and demand/use that makes the co-production of ES. Thus, a methodology that would combine these types of different indicators would be desirable, being an open field for future research. Secondly, some data used in this study are fairly crude and it might miss to capture more fine-grain aspects particularly important for certain services. This, for example, is the case of pollination services that in our study were based on relatively crude land-cover data (see 10.1007/s13280-014-0601-y) as well as a broad classification of suitable habitat. These downsides of the approach should be taken into account when drawing conclusions from our results. That said, and as this approach is increasingly being applied in multiple regions and across different scales, we consider that this methodology has a substantial potential for providing comparable insights on the distribution and interaction of multiple ES, especially when the capacity for conducting more exhaustive assessments is limited.

## Implications for management

Our study demonstrated that a high number and abundance of ES could be found in human-dominated landscapes, which should be considered by management options. In particular, we showed that most cultural services were found in, or near, densely populated urban municipalities. Thus, enhancing the green infrastructure that provides these services in urban areas is likely to provide substantial further benefits, relative to increasing such infrastructure far from the city. Despite the high competition for housing space in a fast growing city as Stockholm, ensuring that housing can co-exist with natural areas would enable the production of ES utilized by large numbers of people.

Identifying bundles of ES can bring numerous benefits for managers and policy makers, when managing complex landscapes (Bennett et al. 2009; Deal et al. 2012). Distinct bundles are likely produced by different sets of social–ecological interactions, and implementing policies targeted for bundles of services, instead of individual services, takes advantage of those interactions. This approach has three clear benefits: First, by highlighting interactions among services, it discourages interventions to enhance single ES, such as carbon sequestration, without thinking about its impact on other services. Second, it helps identifying interventions that can have simultaneously desired effects on multiple ES. Third, by identifying types of ES bundles, decision makers can focus on potential shifts between these groups, and identify where, when, and how such shifts are possible or desirable. In this study, municipalities less typical from each bundle group had likely higher potential for the implementation of management strategies that can lead to a change in the balance of ES.

Our study shows that in the Norrström region in Sweden, there is high potential for multifunctionality of ES at the municipality scale, in particular, in the municipalities around lakes Mälaren and Hjälmaren. Although these are very positive and encouraging findings for regional and local managers and politicians, it is important to notice that trade-offs among services might happen at other spatial scales. Furthermore, multifunctionality might not always be desirable at this scale for all cases (Pasari et al. 2013). For example, the protection of some key iconic species needing large areas of native habitat might imply the existence of contiguous areas of forest, at the cost of some provisioning and cultural services. Such decisions need to be taken based on a broader scale analysis, even knowing they will compromise heterogeneity and multifunctionality at smaller scales. A lot of this knowledge required for management decisions at multiple scales is still lacking and we encourage future research that would replicate the methodology used in this study at other spatial and administrative scales.

## Electronic supplementary material

Below is the link to the electronic supplementary material.

Supplementary material 1 (PDF 748 kb)
