# Supplementary material for: Mapping bundles of ecosystem services reveals distinct types of multifunctionality within a Swedish landscape
Source: Ambio. 2015 Jan 9;44(Suppl 1):89–101. doi: 10.1007/s13280-014-0601-0 (PMC4289005; doi:10.1007/s13280-014-0601-0)
Supplement: Supplementary file 1 — Supplementary material 1 (PDF 748 kb) [file 13280_2014_601_MOESM1_ESM.pdf]

AMBIO

Electronic Supplementary Material

This supplementary material has not been peer reviewed

Mapping bundles of ecosystem services reveals distinct types of multifunctionality within a Swedish landscape

Cibele Queiroz, Megan Meacham, Kristina Richter, Albert V. Norström, Erik Andersson, Jon Norberg, Garry Peterson

Detailed methods for data collection of ecosystem services

Wheat production

We used the agricultural area set aside for wheat production in each municipality as an indicator of this service. Data was retrieved from the Swedish Board of Agriculture National Statistical Database (Jordbruksverket's statistical database) (URL:http://statistik.sjv.se/Database/Jordbruksverket/Markanvandning/JO10SMANV_2012_beskr.pdf) This data is online and publically available. We compiled data for all 62 municipalities for the most recently available year (2012). The total area of wheat production in each municipality was divided by total municipality land area.

Cattle

For assessing this service we used the number of cattle on agricultural holdings per municipality in June 2010. This data was available at the Swedish Board of

Agriculture National Statistical Database (Jordbruksverket's statistical database). URL:http://statistik.sjv.se/PXWeb/Selection.aspx?px_tableid=JO0103G6.px&px_path=Jordbruksverkets%20statistikdatabas__Husdjur__Antal%20husdjur&px_language=sv&px_db=Jordbruksverkets%20statistikdatabas&rxid=5adf4929-f548-4f27-9bc9-78e127837625 The database holds up-to-date data on all cattle existing in Sweden, as cattle owners are obliged to register all animals at the Swedish Board of Agriculture Central Animal Database (CDB). Agricultural holdings refer to activities such as agriculture, stock-farming or horticulture that are undertaken under a single management.

Each agricultural holding fulfilled one of the following criteria: a) a minimum of 2 hectares of cropland b) a minimum of 5 hectares of agricultural land c) engaged in commercial horticulture of at least 2,500 km^2 outdoor area d) engaged in commercial horticulture with a greenhouse area of at least 200 km^2 **or:** owned a herd that any time from 1 January 2010 to 10 June 2010 included e) at least 10 cattle or f) at least 10 sows, or g) at least 50 hogs, or h) at least 20 sheep or i) at least 1 000 poultry.

The total number of cattle in each municipality was weighted by municipality land area.

Sheep and pig production

The data represents the number of pigs and sheep on agricultural holdings per municipality in June 2010. This data was available at the Swedish Board of Agriculture National Statistical Database (Jordbruksverket's statistical database). URL:http://statistik.sjv.se/PXWeb/Selection.aspx?px_tableid=JO0103G6.px&px_path=Jordbruksverkets%20statistikdatabas__Husdjur__Antal%20husdjur&px_language=sv&px_db=Jordbruksverkets%20statistikdatabas&rxid=5adf4929-f548-4f27-9bc9-78e127837625. For sheep and pigs the statistics were based on a survey made to all agricultural holdings in Sweden in June 2010. Agricultural holdings refer to activities as agriculture, stock-farming or horticulture that are undertaken under a single management.

Each agricultural holding fulfilled one of the following criteria: a) a minimum of 2 hectares of cropland b) a minimum of 5 hectares of agricultural land c) engaged in commercial horticulture of at least 2,500 km^2 outdoor area d)

engaged in commercial horticulture with a greenhouse area of at least 200 km^2 **or:** owned a herd that any time from 1 January 2010 to 10 June 2010 included e) at least 10 cattle or f) at least 10 sows, or g) at least 50 hogs, or h) at least 20 sheep or i) at least 1 000 poultry.

The total number of cattle in each municipality was weighted by municipality land area.

Forest products

We defined forest products as products of commercial interest provided by forests such as timber, fiber or paper pulp. Products that can also be provided by non-commercial forests, such as mushrooms or berries, were not included in this analysis.

We used the area of forest used for commercial purposes as an indicator of the amount of forest products. This indicator represents the potential for the supply of forest products and not actual production values. Data was collected from an online database of the Swedish Forestry Agency, URL:http://www.skogsstyrelsen.se/en/AUTHORITY/Statistics/ and represents the area in km^2 of forest used for commercial purposes in each municipality for the most available recent year (2012). Production forest area in each municipality was weighted by total land area in the municipality.

Crop pollination

As an indicator of crop pollination we used the amount of habitats preferred by pollinators that, within a buffer zone of 200m, intersect cropland area. By this we wanted to select the areas where both the source habitat and potential target crops co-exist. The distance of 200m has been referred in previous studies as a reasonable assumption of the distance a pollinator can fly between its source habitat and the feeding areas. For identifying cropland areas and pollinator-preferred habitats we used the Swedish land cover data 2006 (*Marktäckedata*), which is based on the classification of the Corine Land Cover 2006, a land cover classification for all Europe. Data was retrieved from a governmental online database *Lantmäteriet*, URL: https://www.lantmateriet.se/sv/Kartor-och-geografisk-information/Kartor/Geografiska-teman/GSD-Marktackedata/, in

raster format.

We selected the habitat classes preferred by pollinators in the *Märktäckedata* based on expert personal communication. The habitat classes classified as pollinator habitat and their respective code is listed as it follows:

1.1.2.1.2 Urban areas with more than 200 inhabitants and large green areas
1.1.2.2 Urban areas with less than 200 inhabitants
1.1.2.3 Rural areas with open land
1.2.2 Road and rail networks surroundings.
1.3.1.1 Gravel and sandpits
1.4.1 Urban green areas
1.4.2 Sport and recreational areas
2.2.2 Agricultural land: cultivated berries and fruit
2.3.1 Grasslands
3.2.1 Natural grasslands
3.2.2 Moors and heathland
3.3.1 Beaches, dunes, sand
3.3.4 Burnt areas

Raster files were converted to vector format on Quantum GIS geographical information systems software. We intersected the land cover map obtained for all Sweden with the study area, for obtaining an individual land cover map for the Norrström basin. Further, we selected all pollinator habitats and performed a buffer analysis (200m). Finally, we intersected the buffer zones with cropland habitat, obtaining the area in km^2 with high crop pollination potential for all municipalities. The total crop pollination area for each municipality was weighted by total land municipality area.

Standing and running water quality:

Water quality data was retrieved from a publically available database at the Swedish Water Information System (VISS), URL: http://www.viss.lansstyrelsen.se/Exports.aspx?pluginType=StatisticExport&pluginGuid=01E8EEA2-8F6F-4424-B195-74B949C47238

We used data collected during the period 2004-2008 assessing the quality of surface water for both standing (lakes and coastal areas) and running water

(rivers and streams). The data used in this study refers to the ecological status of the water, which evaluates if the quality of the water is suitable to the presence of indicator plant and animal species. The classification of the water quality status is made according with three criteria: 1) biological quality of the water 2) physiological and chemical quality of the water 3) hydro-morphological factors. The biological quality of the water assesses the condition of benthic fauna, fish, macroalgae, macrophytes and plankton communities, through the measurement of specific parameters (detailed information on sampling data collection and the parameters used for assessing the state of each group is available on annexes 1 and 4 of the Swedish Marine and Water Authority's regulations on classification and quality standards regarding surface water (HVMFS 2013:19), URL: https://www.havochvatten.se/hav/vagledning--lagar/foreskrifter/hvmfs/hvmfs-201319.html

The assessment of physicochemical water quality includes the measurement of synthetic (or artificial) and non-synthetic (such as metals) substances, acidification and eutrophication levels, light and oxygen conditions (detailed information available on annexes 2, 5 and 6 of 4 of the Swedish Marine and Water Authority's regulations on classification and quality standards regarding surface water (HVMFS 2013:19), URL: https://www.havochvatten.se/hav/vagledning--lagar/foreskrifter/hvmfs/hvmfs-201319.html. Note that the assessment of the physicochemical water quality is only conducted in those cases where the biological quality status of the water is classified as “good”.

Hydro-morphological water quality indicators include continuity, hydrological regime and morphology. These are only assessed if the biological, physiological and chemical quality of the water is classified as “good” (detailed information on the measurement of hydro-morphological parameters can be found in annex 3 of the Swedish Marine and Water Authority's regulations on classification and quality standards regarding surface water (HVMFS 2013:19), URL: https://www.havochvatten.se/hav/vagledning--lagar/foreskrifter/hvmfs/hvmfs-201319.html

The combination of these three main criteria originates a classification in 5 levels for ecological quality of surface water:

1. High (all three quality criteria are classified as high status)
2. Good (biological and physicochemical criteria are classified as high status but hydro-morphological criteria have a lower classification)
3. Fair (biological criteria are classified as high status while the other two criteria do not fulfill the requirements for high status classification)
4. Bad (none of the three criteria can be classified as "high status")
5. Very bad (none of the three criteria can be classified as "high status")

We used this classification to produce a score for water quality of surface water areas in each municipality ranging from from 1 (very bad status) to 5 (high status). Further, we multiplied the classification given to each water surface by its area, obtaining a weighted water quality classification for each water surface. The final value of water quality attributed to each municipality was the sum of the scores calculated for all water surfaces assessed in that municipality, weighted by the total water surface area in the municipality. This procedure was repeated separately for standing water (lakes, coastal and brackish water) and running water (rivers and streams).

P and N retention

Retention data for both P and N was retrieved from the online database at the Swedish Environmental Emissions Data (SMED), URL:
http://www.smed.se/vatten/data/plc5
Nitrogen (N) retention refers to the proportion of nitrogen pollution from farmland and private sewers separated from the gross load by soils, lakes and streams before reaching the sea. Phosphorous (P) retention refers to the proportion of phosphorous from all phosphorus loads separated from the gross load by soils, lakes and streams before reaching the sea. P and N retention were expressed by the following expression:

*Net load (reaching the sea) = (1 - retention) * Gross load.*

The total P and N retention value obtained for each municipality was weighted by total land municipality area.

Moose hunting

Moose hunting data was retrieved from a database at the County Administrative Board for the most recent available year (2012/2013) and consisted in a) moose hunting zones that existed in our study area in GIS format, URL: www.gis.lst.se and b) number of shootings per moose hunting area in Excel format, URL: www.algdata.se

All data was processed, sorted by hunting area and converted to csv format in order to match the identification numbers for the hunting areas. Further, we made a join between the shooting data and the hunting areas in QGIS software and all the hunting areas of the study region were merged together. We rasterized the shooting data using raster size 3000 pixels and assessed that the size was detailed enough to minimize the errors. Further, mean number of shootings per municipality were calculated by using zonal statistics. This value was further divided by total land area for each municipality.

Summer cottages

Data on summer cottages areas was retrieved for the most recently available year (2010) from the Swedish National Statistics Database SCB, URL: http://www.scb.se/sv_/Hitta-statistik/Statistik-efter-amne/Miljo/Markanvandning/Fritidshusomraden/12934/2010A01/Fritidshusomraden-2010-lansvis/

Data is built on a survey done every five years by the administrative agency for statistics in Sweden (SCB). Summer cottages are classified in the database as areas that consist of at least 50 holiday homes with a maximum of 150 meters between them. Holiday homes considered for this survey are buildings that have taxation code 211, 213 or 221 in the Real Estate Taxation Register:

211 – one or two dwelling unit, undeveloped land for holiday homes

213 – one or two dwelling unit, building value less than SEK 50000

221 – one or two dwelling unit, holiday home

The total summer cottage area in each municipality was weighted by total land municipality area.

Horseback riding

We used number of horses in agricultural holdings for the most recent available year (2010) as an indicator of horseback riding. This data was available at the Swedish Board of Agriculture National Statistical Database (Jordbruksverket statistical database), URL: http://statistik.sjv.se/Database/Jordbruksverket/databasetree.asp

The data is primarily based on a survey conducted in 2010 to agricultural property owners and the numbers correspond to the exact amount of horses hold by agricultural property on the 10th of June 2010. A targeted survey was also conducted at all Swedish riding schools or "similar organizations/companies that offer horseback riding to the public". In addition, data on number of horses and horse holders was collected from animal protection registry (DSK) for 17 municipalities in Stockholm, Gothenburg and Malmö. The response rate was 86% and, theoretically, the populations should not overlap in the results

Agricultural holdings refer to activities such as agriculture, stock-farming

or horticulture that are undertaken under a single management.

Each agricultural holding fulfilled one of the following criteria: a) a minimum of 2 hectares of cropland b) a minimum of 5 hectares of agricultural land c) engaged in commercial horticulture of at least 2,500 km^2 outdoor area d) engaged in commercial horticulture with a greenhouse area of at least 200 km^2 **or:** owned a herd that any time from 1 January 2010 to 10 June 2010 included e) at least 10 cattle or f) at least 10 sows, or g) at least 50 hogs, or h) at least 20 sheep or i) at least 1 000 poultry.

The total number of horses in agricultural holdings in each municipality was weighted by total land area of the municipality.

Outdoor recreation

Outdoor recreation data was based on a dataset containing areas of national interest for outdoor recreation. Data was collected in GIS format for the counties covering the study area URL: www.gis.lst.se. We used areas of outdoor recreation described under chapter 3, section 6 and chapter 4, section 2 of the Swedish environmental law *Miljöbalken*, URL: http://www.riksdagen.se/sv/Dokument-Lagar/Lagar/Svenskforfattningssamling/_sfs-1998-808/

The data was processed in QGIS 2.0.1-Dufour Geographical Information Systems, and the outdoor recreation areas were intersected by municipality borders in order to get a single value for area of outdoor recreation per municipality. The total area of outdoor recreation per municipality was then weighted by total (land and water) municipality area, as outdoor recreation is not only associated to terrestrial areas but also inland water and coastal areas. Proximity to water or coastline generally substantially increases outdoor recreation value of a certain area.

Cross country skiing:

Cross country skiing as a recreation service was assessed through data from a Swedish online public database for cross-country ski tracks by administrative unit URL: http://www.skidspar.se We retrieved the number of stations with

prepared ski tracks for each of the 62 municipalities. The total number of ski stations reported for each municipality was weighted by municipality land area.

Biodiversity appreciation

Data for biodiversity appreciation was collected from the species database *Artaportalen,* URL: http://www.artportalen.se that compiles data on species observation across the country. We used the total number of species observations reported to the database for each one of the 62 municipalities for all years until February 2014 for the following groups: vascular plants, mosses, mushrooms, birds, mammals, amphibians, fish and algae. The number of species reported for each group was summed to obtain a single value for each municipality. Further, we divided the number of reported species for each municipality by the total municipality area.

Data quality considerations: The Swedish *Artportalen* is an open database where any individual can report a species observation. This means that many observations are reported by amateurs, i.e. not following a standard or systematic sampling scheme. Therefore, the number of observations reported within a certain geographical area is not necessarily representative of the actual diversity of species of the area, rather reflecting the level of interest that people living or using the area have on biodiversity.

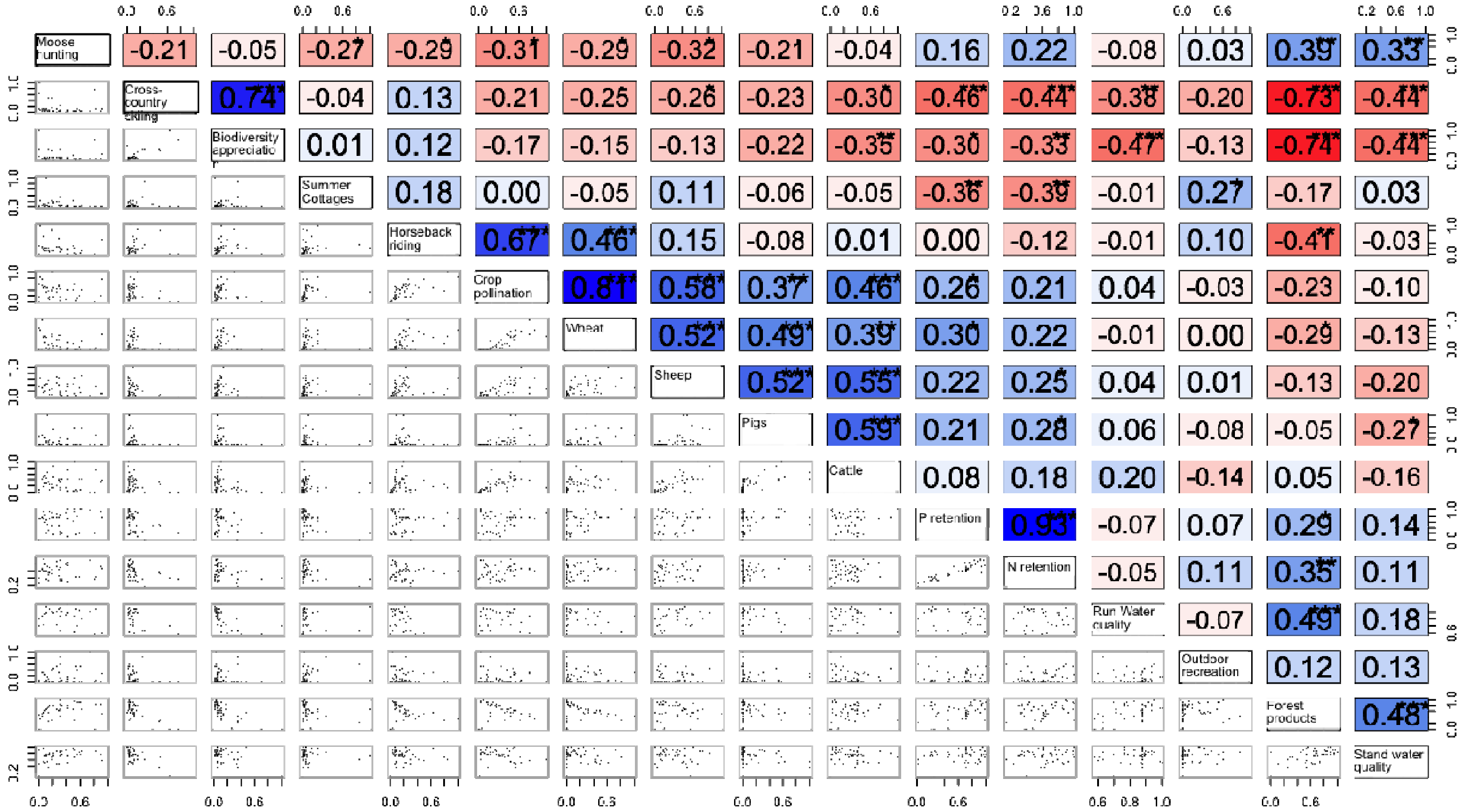

Fig. S1 Results from the correlation analysis of pairs of services with the Pearson correlation test. Blue shadows represent positive correlations and red shadows represent negative correlations. All significant relationships are signalized with *, ** or *** by increasing level of significance.

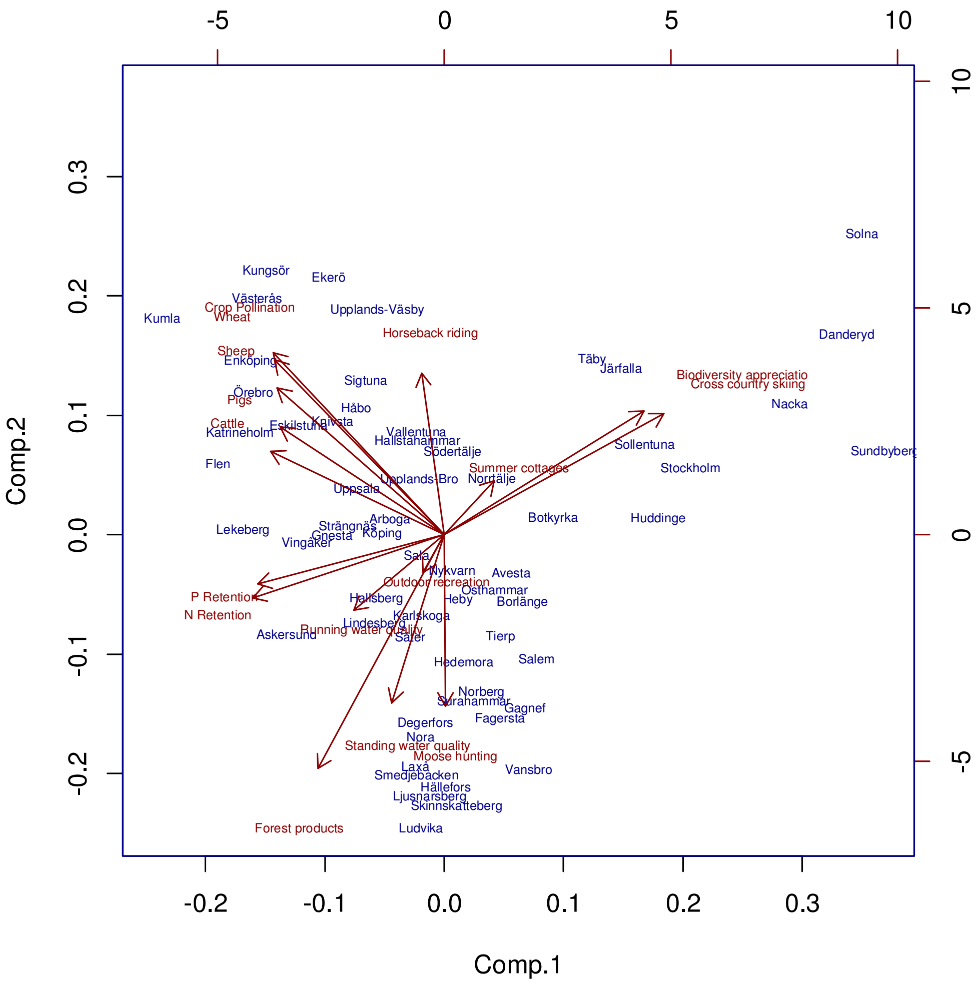

Fig. S2 Principal Component Analysis (PCA)

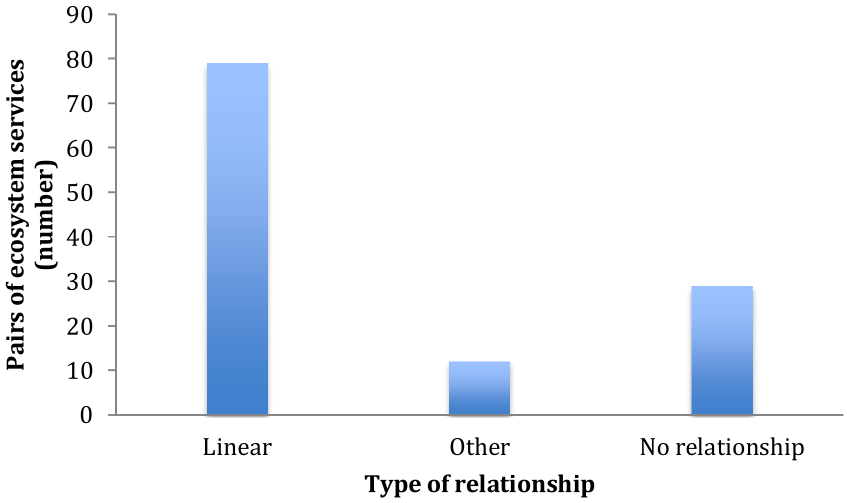

Fig. S3 – Number of pairs of services that present a) a linear relationship b) other types of relationships and c) no relationship
